# H1N1pdm Influenza Infection in Hospitalized Cancer Patients: Clinical Evolution and Viral Analysis

**DOI:** 10.1371/journal.pone.0014158

**Published:** 2010-11-30

**Authors:** Thiago Moreno L. Souza, Jorge I. F. Salluh, Fernando A. Bozza, Milene Mesquita, Márcio Soares, Fernando C. Motta, Melissa Tassano Pitrowsky, Maria de Lourdes Oliveira, Vasiliy P. Mishin, Larissa V. Gubareva, Anne Whitney, Sandra Amaral Rocco, Vânia Maria C. Gonçalves, Venceslaine Prado Marques, Eduardo Velasco, Marilda M. Siqueira

**Affiliations:** 1 Intensive Care Unit, Hospital do Câncer-I/INCA, Rio de Janeiro, Brazil; 2 Laboratório de vírus respiratórios e do sarampo, Instituto Oswaldo Cruz/Fiocruz, Rio de Janeiro, Brazil; 3 Intensive Care Unit, Instituto de Pesquisas Evandro Chagas/Fiocruz, Rio de Janeiro, Brazil; 4 Pediatric Intensive Care Unit, Hospital de Câncer-I/INCA, Rio de Janeiro, Brazil; 5 Infection Control Committee and Infectious Diseases Department, Hospital de Câncer-I/INCA, Rio de Janeiro, Brazil; 6 Influenza Division, National Center for Immunization and Respiratory Diseases/Centers for Disease Control, Atlanta, Georgia, United States of America; National Institute of Environmental Health Sciences, United States of America

## Abstract

**Background:**

The novel influenza A pandemic virus (H1N1pdm) caused considerable morbidity and mortality worldwide in 2009. The aim of the present study was to evaluate the clinical course, duration of viral shedding, H1N1pdm evolution and emergence of antiviral resistance in hospitalized cancer patients with severe H1N1pdm infections during the winter of 2009 in Brazil.

**Methods:**

We performed a prospective single-center cohort study in a cancer center in Rio de Janeiro, Brazil. Hospitalized patients with cancer and a confirmed diagnosis of influenza A H1N1pdm were evaluated. The main outcome measures in this study were in-hospital mortality, duration of viral shedding, viral persistence and both functional and molecular analyses of H1N1pdm susceptibility to oseltamivir.

**Results:**

A total of 44 hospitalized patients with suspected influenza-like illness were screened. A total of 24 had diagnosed H1N1pdm infections. The overall hospital mortality in our cohort was 21%. Thirteen (54%) patients required intensive care. The median age of the studied cohort was 14.5 years (3–69 years). Eighteen (75%) patients had received chemotherapy in the previous month, and 14 were neutropenic at the onset of influenza. A total of 10 patients were evaluated for their duration of viral shedding, and 5 (50%) displayed prolonged viral shedding (median 23, range = 11–63 days); however, this was not associated with the emergence of a resistant H1N1pdm virus. Viral evolution was observed in sequentially collected samples.

**Conclusions:**

Prolonged influenza A H1N1pdm shedding was observed in cancer patients. However, oseltamivir resistance was not detected. Taken together, our data suggest that severely ill cancer patients may constitute a pandemic virus reservoir with major implications for viral propagation.

## Introduction

The emergence of the novel influenza A/H1N1 pandemic virus (H1N1pdm) significantly affected the utilization of healthcare resources and increased morbidity and mortality in children and young adults [1], [2]. From April through September 2009, during the fall/winter in the southern hemisphere, Brazil experienced the first wave of the H1N1pdm virus, and by the end of December 2009, over 1600 H1N1pdm-related deaths had been reported in Brazil [3].

Emerging data on the clinical course of severe H1N1pdm infection have allowed the identification of high-risk groups, which include pregnant women and patients with morbid obesity [4], [5]. However, an analysis of the impact of this novel virus in a highly susceptible population, such as cancer patients, through clinical and virological perspectives, needs to be highlighted [6], [7], [8], [9], [10], [11]. The atypical clinical presentation of influenza infections in cancer patients, which delays clinical suspicion, antiviral treatment and adequate prevention of viral transmission, is a major challenge for clinical management in this population [12]. Cancer patients are more likely to suffer from severe seasonal influenza infections [12], [13], [14] and prolonged viral shedding, as has been reported for an H3N2 seasonal virus [15]. Prolonged shedding and the development of oseltamivir resistance in cancer patients infected with the H1N1pdm virus have not been thoroughly evaluated. Data on these aspects could have major implications for the clinical management and infection control practices for H1N1pdm-infected cancer patients [16].

Because the analysis of this novel viral infection in cancer patients is an important component of the 2009 pandemics, we conducted a prospective cohort study aimed at evaluating the clinical course of influenza infection, the duration of viral shedding, H1N1pdm evolution and the emergence of antiviral resistance in hospitalized cancer patients with a severe H1N1pdm infection in a reference cancer center during the winter of 2009 in Brazil.

## Results

### Characteristics of the study population

**Figure 1:**
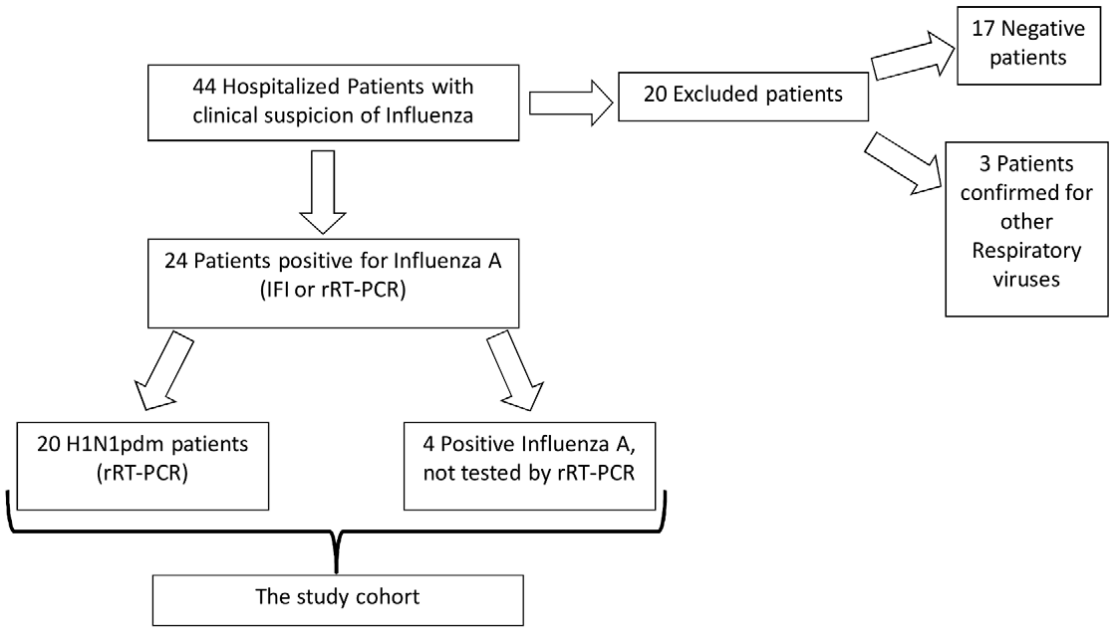
Study flow chart.

During the study period, 44 hospitalized cancer patients with a suspected influenza infection were screened, and 24 had a confirmed influenza A diagnosis using a rapid indirect immunofluorescence (IFI) test or World Health Organization (WHO)-recommended real-time RT-PCR (rRT-PCR) (Figure 1 and Table S1). Among these, 20 patients were confirmed to be positive for the H1N1pdm virus using rRT-PCR (Figure 1 and Table S1). The remaining four patients were positive for influenza A using IFI only. Considering the pandemic case definitions with reference to international guidelines [17], these last four cases were categorized as H1N1pdm-confirmed cases. Altogether, these 24 cases constituted the study population. All of the respiratory samples collected from the 20 rRT-PCR-confirmed patients were inoculated in cell cultures. We recovered the virus from 13 individuals after at least two passages in MDCKs, constituting 15 isolated samples. These isolates were also analyzed for oseltamivir resistance using a functional assay.

Patients diagnosed with H1N1pdm were young (median age  = 14.5, range 3–69 years). In total, 14 (58.3%) were under 18 years old, and 17 (70.8%) were less than 50 years old. Hematologic cancer occurred in 75% (18) of the patients, whereas solid tumors occurred in 25% (6) patients (Tables S2 and S3). A total of 22 (∼92%) patients had received immunosuppressive therapy in the previous 30 days. Among these individuals, 18 patients (75%) were on chemotherapy, 14 (58.3%) received systemic corticosteroids and 1 (4%) received radiation therapy (Table 1 and S4). No patient received erythropoietin (EPO) or immunomodulatory agents. A total of 14 patients (58.3%) presented febrile neutropenia (<500 neutrophils/mm^3^) at the time H1N1pdm was diagnosed. The median duration of neutropenia after the onset of viral disease was two days (ranging from one to six days; Table S5). According to the Brazilian National Cancer Institute's protocol, all patients that presented neutropenia received G-CSF until normalization of neutrophil counts. The clinical characteristics and comparisons among groups are shown in Table 1 and Tables S1, S2, S3, S4, S5, S6, S7, S8, S9, S10, and S11. The overall mortality in our cohort was around 21% (n = 5), and four patients (n = 16.6%) had at least one comorbidity besides cancer. Of these patients with comorbidities, one died. No pregnant or morbidly obese patients were identified.

**Table 1:** Patient characteristics according to survival status^a^.

Variable	All patients (n = 24)	Survivors (n = 19–79.2%)	Non-survivors (n = 5–20.8%)	*P* value b
**Age (years)**	14.5 (3–69)	14 (3–69)	17 (4–62)	0.50
**Male gender**	12 (50%)	7 (36.8%)	5 (100%)	0.03
**Type of cancer**				
*Solid tumor*	6 (25%)	5 (26.3%)	1 (20%)	0.99
*Hematological malignancy*	18 (75%)	14 (73.7%)	4 (80%)	0.99
**Cancer status**				
*Controlled/remission*	3 (12.5%)	3 (15.8%)	0	0.99
*Active - newly-diagnosed*	9 (37.5%)	6 (31.6%)	3 (60%)	0.32
*Active – recurrence/progression*	12 (50%)	10 (52.6%)	2 (40%)	0.99
**Performance status**				
*0–1*	6 (31.6%)	6 (31.6%)	0	0.28
*2–4*	18 (75%)	13 (68.4%)	5 (100%)	0.28
**Previous chemotherapy**	18 (75%)	13 (54.2%)	5 (100%)	0.28
**Previous use of corticosteroids**	14 (58.3%)	10 (52.6%)	4 (80%)	0.36
**Neutropenia**	14 (58.3%)	7 (36.8%)	3 (60%)	0.67
**Need for mechanical ventilation**	10 (41.6%)	5 (26.3%)	5 (100%)	0.06
**Presence of extra-pulmonary organ dysfunction**	8 (33.3%)	4 (21.1%)	4 (80%)	0.03
**Oseltamivir treatment duration (days)**	7 (0–19)	9 (1–19)	5 (1–18)	0.38

a Results are expressed as the mean ± standard deviation, median (range), n (%).

b Reported *P* values refer to comparisons between survivors and non-survivors.

A total of 23 (95.8%) patients were treated with oseltamivir, and the median time from the initial symptoms to the initiation of therapy was three days (0–15 days; Tables 1, S2 and S6). One patient that died due to severe acute respiratory failure 24 h after clinical suspicion of H1N1pdm infection never received antiviral treatment. Oseltamivir was used for a median of seven days (0–19 days), and double doses (150 mg bid for adults and twice the recommended dose per kg for children) were administered for 11 (47.8%) patients (Table S6). A total of 11 (47.8%) patients received oseltamivir for more than seven days. Six patients (25%) received this antiviral within 48 h of clinical suspicion. All patients that died received oseltamivir more than 48 h after the onset of the illness. However, when we compared the mortality of patients that received oseltamivir either within or after 48 h of the onset illness, no significant difference was observed (0/6 vs. 5/18, p = 0.28). No differences in prolonged viral shedding were observed between these two groups.

At presentation, all patients were treated with broad-spectrum intravenous antimicrobial agents to combat community-acquired pneumonia and/or febrile neutropenia [18], and five (20.8%) had concomitant positive cultures (Tables S7 and S8). Hypoxemia was frequent, and the median PaO2/FiO2 on the first arterial blood gas evaluation was 192 mmHg (range: 64–367 mmHg).

### Intensive care unit admission

Overall, 13 patients (five adults and eight children) were admitted to the ICU. Six patients were directly admitted from the emergency department, and the other seven patients were transferred from other hospital wards (Table S2). Ventilatory support was given to 12 patients (Table 1 and S9). Invasive mechanical ventilation was performed in 10 patients (76.9%), and non invasive ventilation (NIV) was performed in 3 patients (23.1%; Table 1). Among the NIV patients, one required subsequent endotracheal intubation and mechanical ventilation, and all three patients were discharged from the hospital. Extra-pulmonary organ failure occurred in eight patients (33.3%; Table 1 and S9).

Of the 13 critically ill patients, 12 were treated with oseltamivir, and treatment was initiated 48 h after the first signs/symptoms of viral infection in 5 of them. Adjunct or non-conventional supportive therapies for ARDS were performed for 12 of the 13 patients that entered the ICU (92.3%). A total of 10 patients (76.9%) received systemic corticosteroids (eight due to previous use and two for shock and persistent ARDS); five (38.5%) were ventilated in a prone position, and four (30.8%) required recruitment maneuvers. No patient received extra-corporeal membrane oxygenation.

### Prolonged H1N1pdm shedding

**Figure 2:**
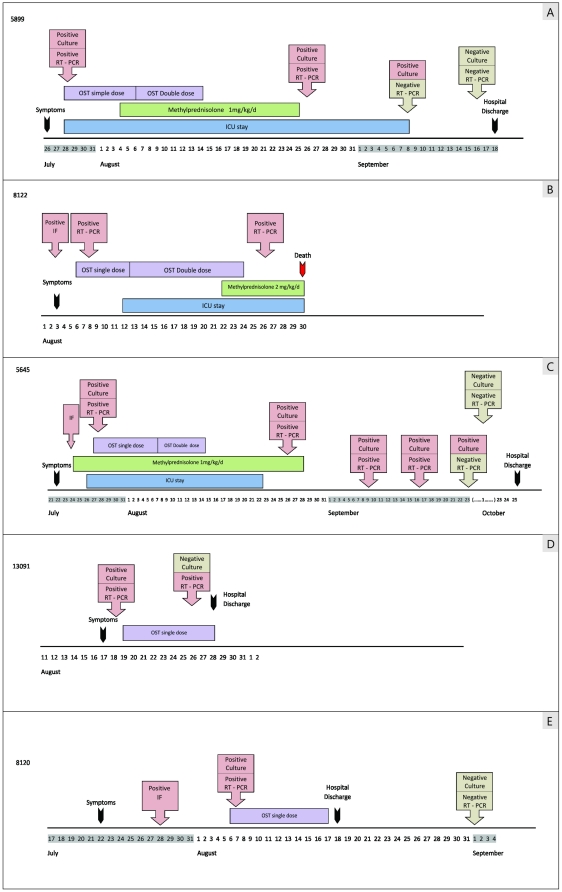
Time course of viral shedding in hospitalized cancer patients.

Although prolonged influenza A shedding has been observed for a cancer patient infected with the H3N2 seasonal virus [15], more detailed data on H1N1pdm secretion in severely ill cancer patients are required. We evaluated viral shedding in 10 mechanically ventilated patients by collecting sequential respiratory samples at different time-points after the onset of illness. The duration of viral shedding was considered to be the time frame from the initial symptoms to the last H1N1pdm-confirmed sample. Five (50%) patients in this group showed viral shedding for at least 11 days during oseltamivir treatment (Figure 2 and Table S12). The median duration of H1N1pdm shedding was 23 days (ranging from 11 to 63 days; Figure 2 and Table S12).

Most importantly, the maximum duration of H1N1pdm shedding in our investigation was 63 days, followed by 44 days for another patient. To our knowledge, these periods constitute the longest registered cases of H1N1pdm shedding described to date. All rRT-PCR-positive samples from these patients with the longest viral shedding durations (5645 and 5899) were culturable, meaning that these were infectious viruses (Figure 2 and Table S12). In addition, the last H1N1pdm-confirmed samples from these patients were only detected using cell culture assays, suggesting the presence of low viral loads in these specimens (Figure 2 and Table S12). These patients still shed the virus for an additional 25 to 40 days after cessation of the antiviral treatment (Figure 2).

### H1N1pdm molecular evolution in clinical isolates

To date, no significant variation has been detected at the amino acid level in the hemagglutinin (HA) of the 2009 pandemic virus [19]. Therefore, we examined the genetic diversity of the H1N1pdm virus recovered from these severely ill patients by performing sequence analysis of the viral HA gene. No significant divergence was found in the H1N1pdm isolates collected during the onset of illness from the four patients with prolonged shedding (Figure S1).

**Figure 3:**
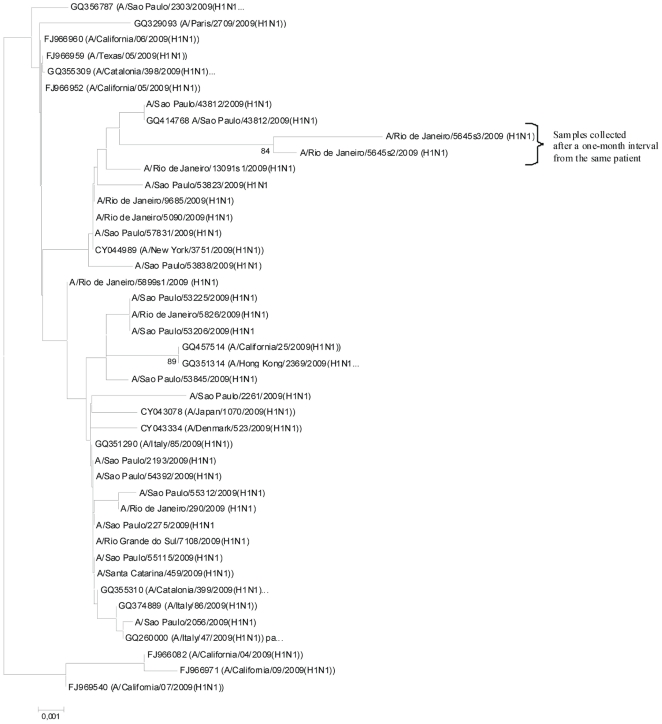
Phylogenetic tree of H1N1pdm strains from HCI-INCa and other Brazilian strains based on the HA gene. The bootstrap probability is indicated for each interior branch, and values below 80% are hidden. The scale bar indicates the number of amino acid changes per site. The sampling number and the state of origin in Brazil are displayed. This tree is rooted by the California/04/2009 HA sequence.

To evaluate the genetic characteristics of the isolates from patients with prolonged viral shedding, we sequenced two consecutive samples from a single individual (5645s2/09 and 5645s3/09) and compared their HA sequences to other H1N1pdm viruses from mild, severe and fatal cases from different countries. We observed that samples collected a month apart (5645s2/09 and 5645s3/09) clustered together and displayed a relatively large branch length from other H1N1pdm viruses (Figure 3). This result may have occurred because strain 5645s2/09 diverged from CA/04 in the amino acid residues L52S, L70P, P100S, C153L, T214A, Q293R and I321V (H1 numbering). Additionally, isolate 5645s3/09, which was sequenced from amino acid residues 167 to 413, also had the mutations T214A, Q293R and I321V fixed in the viral population a month after the initial sampling. In the isolate 5645s3/09, another mutation was also acquired, D238P, suggesting continuous viral evolution. Although we cannot determine the role of these mutations in viral pathogenesis by this result alone, the retention of the changed residues over time strongly suggests viral persistence rather than re-infection.

### H1N1pdm susceptibility to oseltamivir

Considering that influenza resistance to antivirals is likely to emerge in immunocompromised individuals and that, with respect to the H1N1pdm virus, only a few studies have detected oseltamivir resistance [20], we investigated the emergence of antiviral resistance in H1N1pdm samples isolated in cell culture. Thus, oseltamivir carboxylate IC_50_ values were measured for 15 H1N1pdm samples isolated from 13 patients. We found that 10 isolates were sensitive (0.85±0.27 nM), and 5 displayed high IC_50_ values for the antiviral used (165.13±42.26 nM). These samples were sent to the WHO collaborating center at the CDC in Atlanta for resistance confirmation. We found that the isolates with high IC_50_ values were endowed with a neuraminidase (NA) activity that was cross-resistant to oseltamivir, zanamivir and peramivir, suggesting the presence of a co-pathogen endowed with NA activity within these isolates. Co-infections with other respiratory viruses (coronavirus (229, 43 and 63), parainfluenza (1, 2, 3 and 4), human metapneumovirus, parechovirus, rhinovirus, RSV A/B, adenovirus and enterovirus) or atypical bacteria (*Mycobacterium pneumonia*) were not identified in these samples, suggesting that no other viral source of NA activity was responsible for these high IC_50_ values. However, we co-isolated a *Streptococcus* sp. from the samples with high IC_50_ values. The NA activity of this bacterial strain displayed a phenotype resistant to NAIs. In further testing with bacteria-free H1N1pdm isolates, the virus' IC_50_ were consistent with a sensitive isolate. Pyrosequencing analyses were also performed and revealed that the other high-IC_50_ samples had the WT H275 residue and thus did not contain this oseltamivir resistance marker. Viruses isolated from the initial onset of illness were A/California/07-like viruses (Figure S2).

## Discussion

Prolonged influenza shedding in cancer patients has been observed for seasonal strains [15]. Regarding the H1N1pdm virus, it has been shown that prolonged virus shedding in cancer patients may occur, although such a phenomenon has only been documented through cases involving a single studied patient [21], [22], [23], [24], [25]. Here, we prospectively and systematically collected information from a cohort of hospitalized cancer patients with severe H1N1pdm infections. These patients presented high mortality, prolonged viral shedding and H1N1pdm evolution without the emergence of oseltamivir resistance. This is the first study to address viral shedding and resistance in cancer patients with H1N1pdm infections; thus, it may provide insight into the role of cancer patients as potential human reservoirs for this pandemic virus.

Unlike previous reports, our population was composed of hospitalized, severely immunocompromised cancer patients [26]. Most of them were young, had hematologic malignancies and received chemotherapy and systemic steroids in the weeks that preceded the H1N1pdm infection. The patients were treated with oseltamivir in the early course of the infection (the median time to antiviral initiation was three days). A total of 13 patients (54%) required intensive care and presented severe respiratory distress. In these patients, the mortality rates were higher (38%) than those observed for general ICU patients suffering from H1N1pdm infections [2], [27] as well as for non-critically ill cancer patients [26]. However, the outcomes were not different from those reported for cancer patients requiring mechanical ventilation [28], [29].

Interestingly, during the influenza season, 14 patients (58.3%) with febrile neutropenia were identified as H1N1pdm cases, a condition that is not usually investigated in this scenario. However, febrile neutropenic cancer patients have an increased risk of developing respiratory distress and multi-organ failure. Therefore, screening for respiratory viruses and prompt initiation of oseltamivir treatment should be considered in these patients. Febrile neutropenia indicates a poor prognostic with respect to a patient's outcome, but neutropenia duration in our cohort of patients was less than seven days. Thus, prolonged viral shedding might not have a correlation with neutropenia.

We observed the persistence of H1N1pdm in 5 out of 10 patients studied for this purpose. In these individuals, viral shedding continued for at least 11 days, despite the use of oseltamivir. The median duration of viral shedding in our population was 23 days, and two pediatric patients with acute lymphoblastic leukemia showed even longer virus secretions (44 and 63 days; Figure 2 and Table S10), although it is difficult to determine whether viral persistence was due to cancer *per se* or to acute lung injury and mechanical ventilation.

Influenza shedding is not considered to last long, and it disappears seven days after the onset (2.4 and 4.5 days for oseltamivir- and placebo-treated groups, respectively) [30]. Studies aimed at monitoring 2009 H1N1pdm virus shedding using randomized trials with appropriate controls, such as outpatients and hospitalized or immunocompromised individuals, have not yet been conducted. Because we were also unable to establish age-matched controls with or without immunosuppression, since this study was conducted during the peak of the first wave of the 2009 pandemics in Brazil, we compared our work to other studies on H1N1pdm shedding in general, hospitalized or immunocompromised populations [21], [22], [23], [24], [25], [31], [32], [33], [34], [35], [36]. In Table 2, we summarize the cohort used in each study, whether or not they were immunocompromised, their underlying diseases and the number of patients analyzed for viral shedding in each of these studies. We compared the maximum periods of shedding among these different populations and the number of patients that secreted the virus for more than seven days. These data would be more informative and relevant from a public health point of view because the duration of the quarantine for H1N1pdm was approximately that long [21]. We found (Table 2) that in households in Hong Kong [31] and Canada [34], the maximum periods of H1N1pdm shedding ranged from 8 to 11 days. These periods were not different from what was observed with military cadets [22] and during the containment phase of the pandemics in Vietnam [23] (Table 2). Regarding H1N1pdm shedding among infants, Hien et al. showed that children five to nine years old could secrete the H1N1pdm virus for five to six days, which is markedly lower than what is observed for the seasonal influenza virus [30], [33]. Compared to our results, we observed higher periods of viral shedding in two seven-year-old patients with acute lymphoblastic leukemia (Table 2 and Figure 2). However, a single report on two travelers in France showed that these apparently immunocompetent individuals secreted the H1N1pdm virus for 14 and 28 days [25] (Table 2). Although these periods of time are comparable to the time frame of virus secretion in hospitalized patients in China [23] and our work, the study from Felury et al. might be as biased as ours by the small size of the cohort (Table 2). Influenza-infected immunocompromised individuals may have prolonged influenza shedding [15], [21], [32], [36]; however, more insights are necessary to better comprehend the dynamics of the H1N1pdm virus in these individuals. Mora et al. showed that in HIV-1-infected individuals, co-infection with the H1N1pdm virus might lead to an outcome not different from the one expected for immunocompetent subjects, although no systematic analysis of viral shedding was performed [36]. A similar conclusion was also drawn for transplant recipient individuals, whose longest periods of viral shedding did not exceed 11 days [32] (Table 2). In our study, we found periods of H1N1pdm shedding similar to what the CDC observed for leukemia patients [21] (Table 2). Although the small size of these cohorts of immunocompromised individuals [21], [32], including ours, may require a more conclusive and mechanistic analysis, these observations may stimulate further systematic studies to understand or gain insight into factors associated with prolonged H1N1pdm shedding. In addition, it might give insights on basic studies on influenza pathogenesis.

**Table 2:** Comparisons of the period of viral shedding in the general, hospitalized and immunocompromised populations.

	General Population					Hospitalized	Immunocompromised		
**Authors**	**Cowling et al.**	**Hien et al.**	**De Serres et al.**	**Fleury et al.**	**Witkop et al.**	**Cao et al.**	**Seville et al.**	**CDC**	**Ours**
**Reference**	[31]	[33]	[34]	[25]	[22]	[23]	[32]	[21]	
**General population**	Households, Hong Kong	Containment of the pandemics in Ho Chi Minh City (HCMC), Vietnam	Households, Canada	Travelers, France	Military cadets	First 426 patients hospitalized in China	No	No	No
**Immunocompromised**	ND	ND	ND	No	ND	ND	Yes	Yes	Yes
**Underlying disease**	ND	ND	ND	No	ND	Various	Transplant recipients	Leukemia	Cancer
**Number of patients evaluated for virus shedding**	54	932	43	2	29	350	6	2	10
**Number of patients with virus shedding ≥7 days**	2 children	∼80	8–14^b^	2	7	238	1	2	5
**Highest period of shedding (days)**	8	11–12^a^	8–11^b^	14–28^c^	9	17	11	37–44^c^	63

a – Five to six days for children under nine years old.

b – Eight patients shed the virus for at least 8 days, while 14 shed for at least 11 days.

c – Only two patients were evaluated.

ND – Not determined.

Our results highlight the need for closer surveillance of cancer patients with H1N1pdm infections until the detection of the first negative sample. We hypothesize that follow-up protocols aimed at monitoring the persistence of viral shedding in cancer patients may be relevant if patients are submitted to immunosuppressive therapies in the days or weeks prior to or following an H1N1pdm infection.

Next, we sought to determine viral evolution during prolonged shedding. We found that some amino acid changes persisted from the initial symptoms until 30 days thereafter, suggesting that these patients had viral persistence rather than re-infection. In addition, an extra amino acid change (D238P) was found in the viral HA sequenced a month after the onset of illness, suggesting continuing viral evolution. Although some of the mutations that we found (L52S, L70P, P100S, C153L, T214A, D238P, Q293R and I321V) in strains 5645s2/09 and 5645s3/09 have not been previously described, other amino acid residue changes that were detected in our study (P100S and T214A) have been found in H1N1pdm viruses throughout the world without a significant link to viral pathogenesis or antigenic variation [37], [38], [39].

Influenza viruses resistant to antiviral drugs have been reported in immunocompromised patients, [21], [40] and this resistance might be associated with prolonged viral shedding [41], [42]. Notably, five isolates from two patients had high IC_50_ values to neuraminidase inhibitors (NAIs). An NA activity that was multi-resistant to NAIs was identified and could be due to the presence of a *Streptococcus* strain found in throat swabs and tracheal aspirates. Pyrosequencing analyses of samples with high IC_50_ values revealed that these specimens were H275 wild-type sensitive viruses. These results reinforce the need for additional genotyping assays to confirm the identification of putative resistant strains identified using functional assays. In addition, our findings show the need for investigating other sources of NA activity in virus isolates with odd IC_50_ values.

The apparent paradox of prolonged viral shedding without antiviral resistance could be explained by either the inability of the immunocompromised host to effectively clear the H1N1pdm virus [12], [43] or inefficient absorption of the drug [44]. Because we combined both clinical and molecular virology data, our results might contribute to the discussion on the adequate duration and type of anti-H1N1pdm treatment in immunocompromised patients with a protracted course. In these patients, the use of parenteral systemic or inhaled antivirals should also be investigated.

Although our work further investigates the unique dynamics of H1N1pdm virus infection in immunocompromised hosts, some caveats must be noted. Because our investigation started during a new pandemic, the clinical evaluation and management protocols changed during the course of the study as new data emerged from the literature and from updated recommendations [45]. As the pandemic reached its peak in South America, the establishment of a larger and more diverse cohort with age-matched controls with or without immunosuppression became complex. Thus, more in-depth multivariate and mechanistic clinical analyses were limited. Moreover, no recommendations for monitoring viral persistence were available; therefore, only a subset of severely ill patients admitted to the ICU was evaluated. Despite that, an important connection between clinical and laboratory information was studied, revealing the continuous evolution of H1N1pdm HA sequences and the stability of the NA gene in severely ill patients [14].

In conclusion, this study provides evidence that severe H1N1pdm infection is associated with significant morbidity and mortality in cancer patients. In these patients, viral persistence without the emergence of antiviral resistance may occur during the clinical course of the disease. This result has major implications for the clinical management of H1N1pdm infections and infection control strategies. Our study may provide insights into H1N1pdm shedding and might contribute to the development of new guidelines to manage cancer patients with H1N1pdm infection.

## Methods

### Ethics statement

The Ethics Committee (Comitê de Ética em Pesquisa; CEP; http://www.inca.gov.br/conteudo_view.asp?id=2380) at the Instituto Nacional de Câncer (INCa), Rio de Janeiro, Brazil, headed by Dr. Adriana Scheliga approved the study under protocol #18/2010 and waived the need for informed consent.

### Design and setting

This was a prospective cohort study conducted in the Hospital do Câncer-I, Instituto Nacional de Câncer (HC-I-INCa), Rio de Janeiro, Brazil, from July 8^th^ to October 1^st^, 2009. The HCI-INCa is a 160-bed comprehensive cancer center primarily for the population of Rio de Janeiro and neighboring states. The present study was strictly observational, and every clinical decision was at the discretion of the attending physician.

### Patients, data collection and definitions

All patients with a definite diagnosis of cancer requiring hospital admission for any reason and who displayed influenza-like illness were evaluated. Patients in complete remission from cancer for more than five years were not considered.

Data were collected using a standardized case report form that included demographic data, clinical presentation, comorbidities, cancer status, use of immunosuppressive therapies, time course of acute illness, need for intensive care, use of antivirals, adjunctive therapies, advanced life support and in-hospital mortality (supporting information; SI). Patients were included if they had a fever (>37.8°C) and/or respiratory influenza-like illness and confirmed influenza A H1N1pdm diagnosis (by at least one of three assays, IFI, rRT-PCR or cell culture, and according to case definitions from the WHO [46]). Patients were treated according to the Brazilian Public Health guidelines [47].

### Sample collection and analysis

Nasopharyngeal Dacron-swab specimens were collected from all patients and placed onto transport medium (Hanks solution with 100 U/mL penicillin and 100 µg/mL streptomycin) at the initial evaluation. Tracheal aspirates were also obtained if the patient required tracheal intubation. Patients' clinical samples were directly tested for a panel of respiratory viruses using an IFI assay for influenza A (respiratory virus panel; Biotrin, Mount Merrion, Co. Dublin, Ireland.). Specimens were also sent to the Brazilian National Influenza Center (IOC/Fiocruz) for H1N1pdm confirmation using rRT-PCR, which was performed in accordance with the current guidelines from the WHO/CDC [46]. Viral shedding was evaluated in the subset of patients that remained under mechanical ventilation for longer than seven days and in those patients with persistent hypoxemia and pulmonary infiltrate. These patients received a specific number to which their sample number was appended. That is, the first sample was “s1”, and subsequent specimens were numbered consecutively (Table S12). Viral secretion was evaluated using both cell culture and rRT-PCR assays until it was negative. Virus isolation was performed in Madin-Darby canine kidney (MDCK) cells and/or embryonated eggs (see Text S1 and Table S12). The functional antiviral assay was performed using the NA-Star kit (Applied Biosystems, CA), according to the manufacturer's instructions. The RT-PCR protocol for sequencing using the Sanger method and pyrosequencing [48] are presented in the SI, as well as the phylogenetic analysis.

Blood samples were routinely sent for bacterial culturing, as were tracheal aspirates if the patient was intubated.

### Statistical analysis

Standard descriptive statistics were used to describe the study population. Continuous variables were reported as the mean ± standard deviation or median (range) as appropriate. Univariate analysis was used to identify factors associated with hospital mortality. Two-sample *t*-tests and a chi-square or Fisher's exact test were also used. Two-tailed *P* values <0.05 were considered statistically significant.

## Supporting Information

Text S1 Clinical investigation and Influenza virus assays.
(0.06 MB DOC)

Table S1 Diagnostic tests for Influenza A virus.
(0.04 MB DOC)

Table S2 Patient's characteristics and outcomes according to age range.
(0.04 MB DOC)

Table S3 Prevalence of underlying malignancies.
(0.03 MB DOC)

Table S4 Use of Chemotherapy, corticosteroids and granulocyte colony stimulating factor previous to H1N1pdm infection.
(0.04 MB DOC)

Table S5 Patient's Neutropenia.
(0.04 MB DOC)

Table S6 Oseltamivir treatments.
(0.03 MB DOC)

Table S7 Focus of bacterial infection in cancer patients with Influenza A H1N1pdm.
(0.03 MB DOC)

Table S8 Bacteria isolates from cancer patients with Influenza A H1N1pdm.
(0.03 MB DOC)

Table S9 Organ Dysfunctions 72 h after H1N1pdm diagnosis.
(0.03 MB DOC)

Table S10 Frequency of signs and symptoms at clinical suspicion.
(0.03 MB DOC)

Table S11 Pulmonary infiltrates at Influenza diagnosis and hospital discharge, by chest radiography.
(0.03 MB DOC)

Table S12 Clinical and Viral Characteristics of the followed-up cohort.
(0.08 MB DOC)

Figure S1 Phylogenetic tree of HA gene from the classical swine, Eurasian swine, American Avian and human seasonal lineages. The bootstrap probability is indicated for each interior branch, all values below 80% are hidden. The scale bar indicates the number of amino acid changes per site. Colored circles indicate the samples from our study. This tree is unrooted. Each Influenza HA lineage is displayed beside their respective clade.
(0.12 MB TIF)

Figure S2 Phylogenetic tree of NA gene from the followed-up cohort. The bootstrap probability is not indicated for each interior branch since it is below 85%. The scale bar indicates the number of amino acid changes per site. The tree is rooted by California/07/2009 NA sequence.
(0.18 MB TIF)
