# Supplementary material for: H1N1pdm Influenza Infection in Hospitalized Cancer Patients: Clinical Evolution and Viral Analysis
Source: PLoS One. 2010 Nov 30;5(11):e14158. doi: 10.1371/journal.pone.0014158 (PMC2994772; doi:10.1371/journal.pone.0014158)
Supplement: Text S1 — Clinical investigation and Influenza virus assays. (0.06 MB DOC) [file pone.0014158.s001.doc]

**Supporting Information**

**Methods**

Data collected from patients were the following:

- Gender
- Age
- Hospital admission date
- Performance status
- Cancer status (controlled, progression, recurrence)
- Cancer characteristics
  - If solid, extension and primary site
  - If hematologic malignancies, the cellular type
- Cancer treatments 30 days prior to admission (radiotherapy, chemotherapy and corticosteroids)
- Comorbidities besides cancer (Charlson criteria)
- Clinical data from influenza A H1N1pdm disease
  - Initial symptoms and duration
  - Time of symptom presentation before clinical suspicion and treatment initiation
  - Radiological images at clinical suspicion and disease progression
  - Immunofluorescence results for influenza A and B
  - Data from reverse transcriptase-polymerase chain reaction for H1N1pdm in nasopharyngeal swabs and tracheal secretions (for intubated patients)
  - Laboratory data (blood cell count, electrolytes, arterial blood gases, renal and hepatic function, muscular enzymes and C-reactive protein)
  - Presence and duration of neutropenia
  - Antiviral treatment (oseltamivir dose and duration)
  - Corticoisteroid use during influenza treatment
- Data from ICU admission (when appropriate)
- Cause for admission
- Date and length of stay
- Organ dysfunction after 24 h
- Bacterial infection (focus and severity)
- Need for mechanical ventilation (non-invasive, invasive, duration)
- Need for adjunctive therapies for ARDS (prone position, recruitment and corticosteroids)
- Renal impairment and dialysis
- Outcome
- Length of hospital stay
- Outcomes and mortality

**Influenza virus assays**

**Cell preparation**. Madin-Darby Canine Kidney (MDCK) cells were cultured in Dulbecco’s modified Eagle’s medium (DMEM; GIBCO, Grand Island, NY) supplemented with 10% fetal bovine serum (FBS; Hyclone, Logan, Utah), 100 U/mL penicillin and 100 µg/mL streptomycin and were incubated at 37°C in 5% CO_2_ [1].

**Virus isolation.** To isolate infectious viruses, clinical samples were filtered through 0.22 µm filters and were inoculated in either embryonated eggs or MDCK cells. On day 9, the embryonated eggs were inoculated with the clinical samples through the allantoic route. Viral growth was stopped five days after inoculation and allantoic fluid was collected [1].

MDKCs were washed three times with DMEM containing 4 µg/mL trypsin and 0.2% serum albumin (inoculation medium). After that, they were incubated with 100–200 µL of the clinical samples in individual tubes in duplicate for 1 h at 35°C. Next, we added an additional 0.8 mL of inoculation medium per tube. The cells were microscopically observed for cytopathic effects each day, up to the seventh day after infection, and after this period, their supernatants were harvested [1]. We confirmed viral isolation using hemagglutination, neuraminidase or rRT-PCR assays [1,2].

Viruses were passaged no more than three times.

**RNA extraction** Total RNA was extracted from clinical samples, the supernatant of the cell cultures or allantoic fluid. RNA extraction was performed using the viral RNA mini kit (Qiagen, CA), according to the manufacturer’s instructions. RNA was eluted in TE buffer (pH 8.0) and stored at -70°C.

**H1N1pdm detection**. Real-time RT-PCR was performed as a one-step assay with the enzyme Superscript III and Platinum Taq. To confirm H1N1pdm cases, we followed the WHO/CDC Atlanta protocol for detection of the influenza genes M, NP and HA and the housekeeping gene RNase P. The cycling conditions were 50°C for 30 minutes and 95°C for 2 minutes followed by 45 cycles of 92°C for 15 seconds and 55°C for 35 seconds [2].

**Respiratory virus and atypical bacteria detection.** Samples and isolates from two patients (5645 and 5899) were tested for the presence of respiratory viruses (coronavirus (229, 43 and 63), parainfluenza (1, 2, 3 and 4), human metapneumovirus, parechovirus, rhinovirus, RSV A/B, adenovirus and enterovirus) and atypical bacteria (*Mycobacterium pneumonia*) using a one-step real-time RT-PCR assay with the enzyme Superscript III and Platinum Taq, according to the manufacturer’s instructions (Fast-Track diagnosis, Luxembourg).

**Antiviral assay*.*** To determine the IC_50_ values of our samples to oseltamivir carboxylate, we performed functional antiviral assays using the NA-star kit (Applied Biosystems), according to the manufacturer’s instructions. In brief, the viral isolates obtained from MDCKs or embryonated eggs were centrifuged at 800 x g for 10 min to remove cellular debris. Next, the neuraminidase (NA) activities in these samples were titered as a two-fold dilution to determine the working dilution of the virus. After that, the NA activity of the viral isolates was measured in the presence of different concentrations of oseltamivir carboxylate. For comparison, assays with wild-type and resistant standard strains of influenza A and B were also performed. Pyrosequencing assays were performed as previously described [3].

**Sequencing and phylogenetic analysis*.*** To monitor viral evolution, we analyzed H1N1pdm HA and NA genes. First, the RNA extracted from either clinical samples or viral isolates were subjected to one-step RT-PCR using Superscript III and Platinum Taq and previously described primers [4]. The RT-PCR products were purified, and sequencing was performed using the Sanger method, using the “Big Dye Terminator Cycle Sequencing Ready Reaction” kit (Applied Biosystems). After that, fragments were analyzed using an automatic sequencer (ABI PRISMTM 3100-avant Genetic Analyzer; PE Applied Biosystems). Pyrosequencing assays were performed as previously described [3].

Consensus sequences for both HA and NA genes were generated in SeqEd (Applied Biosystems) and aligned to other sequences deposited in GenBank using the ClustalW algorithm in Megalign (Mega software 4.0). Sequences were analyzed using neighbor-joining with bootstrap (1,000 times) using the Mega 4.1 suite of programs. Bootstrapping is a statistical sampling technique for providing estimates of how often permutations of the dataset support the branching order of the phylogenetic tree and indicates the confidence with which the tree can be interpreted. Bootstrap values greater than 80 were considered significant.

**References**

1. Szretter KJ, Balish AL, Katz JM (2006) Influenza: propagation, quantification, and storage. Curr Protoc Microbiol Chapter 15: Unit 15G 11. DOI:10.1002/0471729256.mc15g01s3

2. World Health Organization (2009) CDC protocol of realtime RTPCR for influenza A(H1N1). Available: http://www.who.int/csr/resources/publications/swineflu/CDCRealtimeRTPCR_SwineH1Assay-2009_20090430.pdf. Accessed 2009 April 30

3. Deyde VM, Sheu TG, Trujillo AA, Okomo-Adhiambo M, Garten R, et al. (2010) Detection of molecular markers of drug resistance in 2009 pandemic influenza A (H1N1) viruses by pyrosequencing. Antimicrob Agents Chemother 54: 1102-1110.

4. World Health Organization (2009) Protocol of virus sequencing. Available: http://www.who.int/csr/resources/publications/swineflu/GenomePrimers_20090512.pdf. Accessed 2009 May 12
