# Supplementary material for: H1N1pdm Influenza Infection in Hospitalized Cancer Patients: Clinical Evolution and Viral Analysis
Source: PLoS One. 2010 Nov 30;5(11):e14158. doi: 10.1371/journal.pone.0014158 (PMC2994772; doi:10.1371/journal.pone.0014158)
Supplement: Table S1 — Diagnostic tests for Influenza A virus. (0.04 MB DOC) [file pone.0014158.s002.doc]

**Table S1 - Diagnostic tests for Influenza A virus**

| **Patient** | IFI* | **RT- PCR**** |
| --- | --- | --- |
| 1 | Positive | Not Performed |
| 2 | Positive | Positive |
| 3 | Positive | Positive |
| 4 | Positive | Positive |
| 5 | Positive | Not Performed |
| 6 | Positive | Not Performed |
| 7 | Positive | Positive |
| 8 | Positive | Positive |
| 9 | Positive | Positive |
| 10 | Positive | Not Performed |
| 11 | Positive | Positive |
| 12 | Negative | Positive |
| 13 | Positive | Positive |
| 14 | Positive | Positive |
| 15 | Not Performed | Positive |
| 16 | Negative | Positive |
| 17 | Not Performed | Positive |
| 18 | Positive | Positive |
| 19 | Positive | Positive |
| 20 | Positive | Positive |
| 21 | Negative | Positive |
| 22 | Positive | Positive |
| 23 | Positive | Positive |
| 24 | Positive | Positive |

*IFI detects influenza A.

**RT-PCR detects influenza A H1N1pdm vírus.
