# Supplementary material for: H1N1pdm Influenza Infection in Hospitalized Cancer Patients: Clinical Evolution and Viral Analysis
Source: PLoS One. 2010 Nov 30;5(11):e14158. doi: 10.1371/journal.pone.0014158 (PMC2994772; doi:10.1371/journal.pone.0014158)
Supplement: Table S2 — Patient's characteristics and outcomes according to age range. (0.04 MB DOC) [file pone.0014158.s003.doc]

**Table S2 - Patient’s characteristics and outcomes according to age range^a^**

| **Variables** | **All patients** | **Adults (n = 10 – 41.6%)** | **Children.(n = 14 – 58.%)** | ***P* value ^b^** |
| --- | --- | --- | --- | --- |
| **Median age ( years)^a^** | 14.5 (3 – 69) | 57.5 (19 – 69) | 8 (2 – 17) | 0.002 |
| **Male gender** | 12 (50%) | 3 (30%) | 9 (64.3%) | 0.21 |
| **Female gender** | 12 (50%) | 7 (70%) | 5 (35.7%) | 0.21 |
| **Type of cancer** |  |  |  |  |
| *Solid tumor* | 6 (25%) | 2 (20%) | 4 (28.6%) | 0.99 |
| *Hematological malignancy* | 18 (75%) | 8 (80%) | 10 (71.4%) | 0.99 |
| **Cancer status** |  |  |  |  |
| *Controlled / remission* | 3 (12.5%) | 3 (30%) | 0 | 0.06 |
| *Active - newly-diagnosed* | 9 (37.5%) | 1 (10%) | 8 (57.1%) | 0.03 |
| *Active - recurrence / progression* | 12 (50%) | 6 (60%) | 6 (42.8%) | 0.68 |
| **Performance status** |  |  |  |  |
| *0 - 1* | 6 (25%) | 5 (50%) | 1 (7.14%) | 0.05 |
| *2 - 4* | 18 (75%) | 5 (50%) | 13 (92.8%) | 0.05 |
| **Median duration of Oseltamivir use (days)^a^** | 7 (0 -19) | 5 (0 -10) | 14 (1-19) | 0.05 |
| **Need for ICU admission** | 13 (54.2%) | 5 (50%) | 8 (57.1%) | 0.94 |
| **Median ICU length of stay (days)^a^** | 12 (1 - 44) | 5 (1 - 44) | 13.5 (4 - 35) | 0.88 |
| **Acute respiratory failure** | 14 (58.3%) | 5 (50%) | 9 (64.3%) | 0.68 |
| **Hospital Mortality** | 5 (20.8%) | 2 (20%) | 3 (21.4%) | 0.99 |

^a^ Results expressed as mean ± standard deviation, median (range), n (%).

^b^ Reported *P* values refer to comparisons among adults and children.

ICU = intensive care unit.
