# Supplementary material for: H1N1pdm Influenza Infection in Hospitalized Cancer Patients: Clinical Evolution and Viral Analysis
Source: PLoS One. 2010 Nov 30;5(11):e14158. doi: 10.1371/journal.pone.0014158 (PMC2994772; doi:10.1371/journal.pone.0014158)
Supplement: Table S3 — Prevalence of underlying malignancies. (0.03 MB DOC) [file pone.0014158.s004.doc]

**Table S3 - Prevalence of underlying malignancies.**

| **Underlying malignancies** | **N (%)** |
| --- | --- |
| Acute leukemia | 7 (29%) |
| Chronic leukemia | 2 (8%) |
| Lymphoma | 6 (25%) |
| Multiple Myeloma | 3 (12%) |
| Solid tumors | 6 (25%) |
