# Supplementary material for: H1N1pdm Influenza Infection in Hospitalized Cancer Patients: Clinical Evolution and Viral Analysis
Source: PLoS One. 2010 Nov 30;5(11):e14158. doi: 10.1371/journal.pone.0014158 (PMC2994772; doi:10.1371/journal.pone.0014158)
Supplement: Table S4 — Use of Chemotherapy, corticosteroids and granulocyte colony stimulating factor previous to H1N1pdm infection. (0.04 MB DOC) [file pone.0014158.s005.doc]

**Table S4 - Use of Chemotherapy, corticosteroids and granulocyte colony stimulating factor previous to H1N1pdm** infection

| **Patient** | **Chemotherapy** | **Neutropenia** | **Corticosteroids** | G-CSF |
| --- | --- | --- | --- | --- |
| 24 | Vincristine, Cytarabine, Etoposide, Ifosfamide, Methotrexate | Yes | Yes | Yes |
| 10 | Cyclophosphamide, Vincristine, Methotrexate, Doxorubicin, Cytarabine, Etoposide | Yes | Yes | Yes |
| 22 | Etoposide, Cytarabine | Yes | No | Yes |
| 13 | Etoposide, Cyclophosphamide | Yes | Yes | Yes |
| 4 | Cyclophosphamide, Cytarabine, Methotrexate | Yes | No | Yes |
| 14 | Doxorubicin, Cisplatin | Yes | Yes | Yes |
| 1 | Vincristine, Daunorubicin | Yes | Yes | Yes |
| 8 | Cytarabine, Vincristine, Methotrexate, PEG-L-asparaginase, Daunorubicin, Cyclophosphamide | Yes | No | Yes |
| 9 | Etoposide, Ifosfamide | Yes | No | Yes |
| 5 | Vincristine, Doxorubicin, Methotrexate, Teniposide, Ifosfamide, Cytarabine, Cyclophosphamide | Yes | Yes | Yes |
| 15 | Methotrexate, Doxorubicin | Yes | No | Yes |
| 20 | Dexamethasone | No | Yes | No |
| 2 | Methotrexate, Ifosfamide, Cytarabine, Vincristine, Cyclophosphamide, Mitoxantrone | No | Yes | No |
| 12 | Cytarabine | No | No | No |
| 21 | Rituximab, Cyclophosphamide, Doxorubicin, Vincristine | No | No | No |
| 19 | Vinorelbine, Vinblastine, Methotrexate, Mitomycin | No | No | No |
| 16 | Cytarabine | No | Yes | No |
| 7 | Vincristine, Methotrexate, Ifosfamide, Daunorubicin, Vincristine | No | No | No |
| 23 | nill | Yes | Yes | No |
