# Supplementary material for: H1N1pdm Influenza Infection in Hospitalized Cancer Patients: Clinical Evolution and Viral Analysis
Source: PLoS One. 2010 Nov 30;5(11):e14158. doi: 10.1371/journal.pone.0014158 (PMC2994772; doi:10.1371/journal.pone.0014158)
Supplement: Table S5 — Patient's Neutropenia. (0.04 MB DOC) [file pone.0014158.s006.doc]

**Table S5** - **Patient’s** **Neutropenia**

| **Patient** | **30 days before hospital admission** | **Duration of neutropenia (days)** | **Neutropenia at clinical suspicion** |
| --- | --- | --- | --- |
| 1 | Yes | 6 | Yes |
| 2 | No |  | Yes |
| 3 | Yes | 6 | Yes |
| 4 | Yes | 1 | Yes |
| 5 | Yes | 5 | Yes |
| 6 | No |  | No |
| 7 | No |  | No |
| 8 | Yes | 2 | Yes |
| 9 | No |  | Yes |
| 10 | Yes | 1 | Yes |
| 11 | Yes | 2 | No |
| 12 | No |  | No |
| 13 | No |  | Yes |
| 14 | No |  | Yes |
| 15 | Yes | 1 | Yes |
| 16 | No |  | No |
| 17 | No |  | No |
| 18 | No |  | No |
| 19 | No |  | No |
| 20 | No |  | No |
| 21 | No |  | No |
| 22 | Yes | 1 | Yes |
| 23 | Yes | 1 | Yes |
| 24 | Yes | 2 | Yes |
