# Supplementary material for: H1N1pdm Influenza Infection in Hospitalized Cancer Patients: Clinical Evolution and Viral Analysis
Source: PLoS One. 2010 Nov 30;5(11):e14158. doi: 10.1371/journal.pone.0014158 (PMC2994772; doi:10.1371/journal.pone.0014158)
Supplement: Table S6 — Oseltamivir treatments. (0.03 MB DOC) [file pone.0014158.s007.doc]

**Table S6 - Oseltamivir treatments**

| Antiviral treatment | **N (%)** |
| --- | --- |
| Time for therapy initiation after clinical suspicion (median) | 3 days (range 0 – 15 days) |
| Duration of therapy (median – days) | 7 (range 0- 19) |
| Double doses of oseltamivir | 11 (47.8%) |
