# Supplementary material for: H1N1pdm Influenza Infection in Hospitalized Cancer Patients: Clinical Evolution and Viral Analysis
Source: PLoS One. 2010 Nov 30;5(11):e14158. doi: 10.1371/journal.pone.0014158 (PMC2994772; doi:10.1371/journal.pone.0014158)
Supplement: Table S7 — Focus of bacterial infection in cancer patients with Influenza A H1N1pdm. (0.03 MB DOC) [file pone.0014158.s008.doc]

**Table S7 - Focus of bacterial infection in cancer patients with Influenza A H1N1pdm**

| **Number of patients with associated bacterial infections (site of infection)** | **N (%)** |
| --- | --- |
| Total | 5 (20.8%) |
| Respiratory tract | 1 (20%) |
| Gastrointestinal tract | 1 (20%) |
| Urinary tract | 2 (40%) |
| Bloodstream infection | 1 (20%) |
