# Supplementary material for: H1N1pdm Influenza Infection in Hospitalized Cancer Patients: Clinical Evolution and Viral Analysis
Source: PLoS One. 2010 Nov 30;5(11):e14158. doi: 10.1371/journal.pone.0014158 (PMC2994772; doi:10.1371/journal.pone.0014158)
Supplement: Table S8 — Bacteria isolates from cancer patients with Influenza A H1N1pdm. (0.03 MB DOC) [file pone.0014158.s009.doc]

**Table S8 - Bacteria isolates from cancer patients with Influenza A H1N1pdm**

| **Number of patients with associated bacterial infections (bacteria identification)** | **N (%)** |
| --- | --- |
| Number of isolates | 4 |
| *Pseudomonas aeruginosa* | 2 |
| *Escherichia coli* | 1 |
| *Clostridium difficile* | 1 |
| *Moraxella catarrhalis* | 1 |
