# Supplementary material for: H1N1pdm Influenza Infection in Hospitalized Cancer Patients: Clinical Evolution and Viral Analysis
Source: PLoS One. 2010 Nov 30;5(11):e14158. doi: 10.1371/journal.pone.0014158 (PMC2994772; doi:10.1371/journal.pone.0014158)
Supplement: Table S9 — Organ Dysfunctions 72 h after H1N1pdm diagnosis. (0.03 MB DOC) [file pone.0014158.s010.doc]

**Table S9 - Organ Dysfunctions 72 h after H1N1pdm diagnosis**

| **Patients with organ dysfunction** | **N = 18 (75%)** |
| --- | --- |
| Acute renal insufficiency | 4 (22.2%) |
| Hepatic dysfunction | 2 (11.1%) |
| Respiratory distress (NIV, Oxygen supplementation or mechanical ventilation) | 14 (77.8%) |
| Shock | 8 (44.4%) |
| Thrombocytopenia (< 150.000/mm^3^) | 14 (77.8%) |
| Mean number of associated organ dysfunctions | 2.8 ( ± 1.5) |
| 1 dysfunction | 4 (22.2%) |
| 2 dysfunctions | 6 (33.3%) |
| 3 dysfunctions | 2 (11.1%) |
| 4 dysfunctions | 2 (11.1%) |
| 5 dysfunctions | 4 (22.2%) |
