# Supplementary material for: H1N1pdm Influenza Infection in Hospitalized Cancer Patients: Clinical Evolution and Viral Analysis
Source: PLoS One. 2010 Nov 30;5(11):e14158. doi: 10.1371/journal.pone.0014158 (PMC2994772; doi:10.1371/journal.pone.0014158)
Supplement: Table S10 — Frequency of signs and symptoms at clinical suspicion. (0.03 MB DOC) [file pone.0014158.s011.doc]

**Table S10 - Frequency of signs and symptoms at clinical suspicion**

| **Signs and symptoms** | **N (%)** |
| --- | --- |
| Fever | 23 (95.8%) |
| Cough | 19 (79.2%) |
| Dyspnea | 14 (58.3%) |
| Fatigue | 14 (58.3%) |
| Headache | 5 (20.8%) |
| Sore throat | 4 (16.7%) |
| Nausea/vomiting | 2 (8.3%) |
| Others | 7 (29.1%) |
