# Supplementary material for: H1N1pdm Influenza Infection in Hospitalized Cancer Patients: Clinical Evolution and Viral Analysis
Source: PLoS One. 2010 Nov 30;5(11):e14158. doi: 10.1371/journal.pone.0014158 (PMC2994772; doi:10.1371/journal.pone.0014158)
Supplement: Table S11 — Pulmonary infiltrates at Influenza diagnosis and hospital discharge, by chest radiography. (0.03 MB DOC) [file pone.0014158.s012.doc]

**Table S11 - Pulmonary infiltrates at Influenza diagnosis and hospital discharge, by chest radiography**

| Pulmonary infiltrates | **Number of Patients (%)** |
| --- | --- |
| Absent | 2 (8.3%) |
| Unilateral | 7 (29.2%) |
| Bilateral | 15 (62.5%) |
| Persistent pulmonary infiltrates at hospital discharge | 14 (58.3%) |
