# Supplementary material for: H1N1pdm Influenza Infection in Hospitalized Cancer Patients: Clinical Evolution and Viral Analysis
Source: PLoS One. 2010 Nov 30;5(11):e14158. doi: 10.1371/journal.pone.0014158 (PMC2994772; doi:10.1371/journal.pone.0014158)
Supplement: Table S12 — Clinical and Viral Characteristics of the followed-up cohort. (0.08 MB DOC) [file pone.0014158.s013.doc]

**Table S12 - Clinical and Viral Characteristics of the followed-up cohort**

| **Patient** | **Sample IDa** | **Collection Date** | **Clinical Materialb** | **H1N1pdm Statusc** | **Virus Shedding (days)** | **Passage history** | | **Oseltamivir IC50d (nM)** | **Amino acid residue at position 275 of the NA** | **Type of cancer** | **Clinical outcome (date)** |
| --- | --- | --- | --- | --- | --- | --- | --- | --- | --- | --- | --- |
|  |  |  |  |  |  | **# 1 MDCK** | **# 2 MDCK** |  |  |  |  |
| A | 5644s1 | 28-jul-09 | NFA | POS | NA | POS | POS | 3.2 ± 0.30 | H | NHL | Discharged (4-aug-09) |
|  | 5644s2 | 9-jan-09 | NFA | NEG |  | NA | NA | NA |  |  |  |
| B | 5645s1* | 24-jul-09 | NFA | POS | 63 | NE | NE | NE |  | ALL | Discharged, (9-nov-09) |
|  | 5645s2 | 27-jul-09 | NFA | POS |  | POS | POS | 0.68 ± 0.02 | H |  |  |
|  | 5645s3 | 26-aug-09 | NFA | POS |  | POS | POS | 72 ± 5.0 | H |  |  |
|  | 5645s4 | 8-sept-09 | NFA | POS |  | POS | POS | 94 ± 13 | H |  |  |
|  | 5645s5 | 16-sept-09 | NFA | POS |  | POS | POS | 159 ± 27 | H |  |  |
|  | 5645s6 | 23-sept-09 | NFA | NEG |  | NEG | POS | NE |  |  |  |
|  | 5645s7 | 1-out-09 | NFA | NEG |  | NA | NA | NA |  |  |  |
| C | 5899s1 | 28-jul-09 | TA | POS | 44 | POS | POS | 0.7 ± 0.06 | H | ALL | Discharged (18-sept-09) |
|  | 5899s2 | 26-aug-09 | TA | POS |  | POS | POS | 312 ± 58 | H |  |  |
|  | 5899s3 | 8-sept-09 | TA | NEG |  | NEG | POS | 188 ± 31 | H |  |  |
|  | 5899s4 | 16-sept-09 | TA | NEG |  | NA | NA | NA |  |  |  |
| D | 8120s1 | 28-jul-09 | NFA | POS | 16 | NE | NE | NE |  | ALL | Discharged (18-aug-09) |
|  | 8120s2 | 7-aug-09 | NFA | POS |  | NEG | NEG | NA |  |  |  |
|  | 8120s3 | 1-sept-09 | NFA | NEG |  | NA | NA | NA |  |  |  |
| E | 7301s1 | 3-aug-09 | TA | POS | NA | NEG | NEG | NA |  | MM | Discharged (26-sept-09) |
|  | 7301s2 | 25-aug-09 | TA | NEG |  | NA | NA | NA |  |  |  |
| F | 8122s1* | 3-aug-09 | NFA | POS | 23 | NE | NE | NA |  | NHL | Deceased (30-aug-09) |
|  | 8122s2 | 7-aug-09 | TA | POS |  | POS | NE | 0.4 ± 0.05 | H |  |  |
|  | 8122s3 | 26-aug-09 | TA | POS |  | NEG | NEG | NA |  |  |  |
| G | 13091s1 | 18-aug-09 | NFA | POS | 11 | POS | POS | 0.6 ± 0.03 | H | CML | Discharged (28-aug-09) |
|  | 13091s2 | 26-aug-09 | NFA | POS |  | NEG | NEG | NA |  |  |  |
| H | 15117s1 | 1-sept-09 | TA | POS | NA | NEG | NEG | NA |  | MM | Discharged (22-out-09) |
|  | 15117s2 | 1-oct-09 | TA | NEG |  | NA | NA | NA |  |  |  |
| I | 1560s1* | 29-jul-09 | NFA | POS | NA | NEG | NEG | NA |  | AML | Discharged (11-aug-09) |
|  | 15160s2 | 1-sept-09 | NFA | NEG |  | NA | NA | NA |  |  |  |
| J | 15161s1* | 6-aug-09 | NFA | POS | NA | POS | POS | NE |  | NHL | Discharged (19-aug-09) |
|  | 15161s2 | 1-sept-09 | NFA | NEG |  | NA | NA | NA |  |  |  |

a –The first samples is assigned as “s1” and the subsequent specimens numbered henceforward.

b  - NFA Nasopharyngeal aspirate, TA – tracheal aspirate.

c –  H1N1pdm status is based on the real time RT-PCR results or IFI (*)

d – IC50­ values stand for mean ± SEM

NA – Not Applicable

NE – Not Evaluated
