# Supplementary figures and images for: H1N1pdm Influenza Infection in Hospitalized Cancer Patients: Clinical Evolution and Viral Analysis
Source: PLoS One. 2010 Nov 30;5(11):e14158. doi: 10.1371/journal.pone.0014158 (PMC2994772; doi:10.1371/journal.pone.0014158)

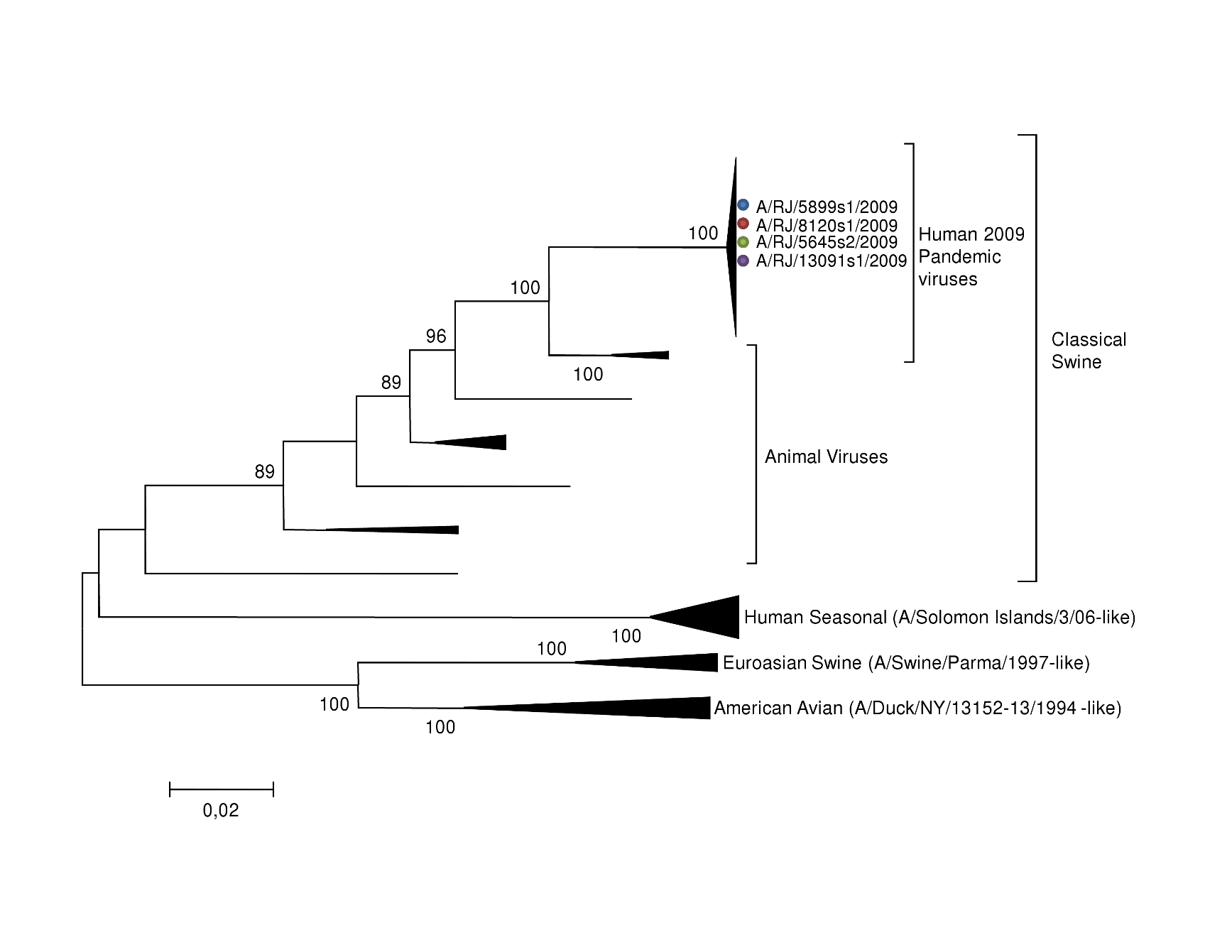

Supplement: Figure S1 — Phylogenetic tree of HA gene from the classical swine, Eurasian swine, American Avian and human seasonal lineages. The bootstrap probability is indicated for each interior branch, all values below 80% are hidden. The scale bar indicates the number of amino acid changes per site. Colored circles indicate the samples from our study. This tree is unrooted. Each Influenza HA lineage is displayed beside their respective clade. (0.12 MB TIF) [file pone.0014158.s014.tif]

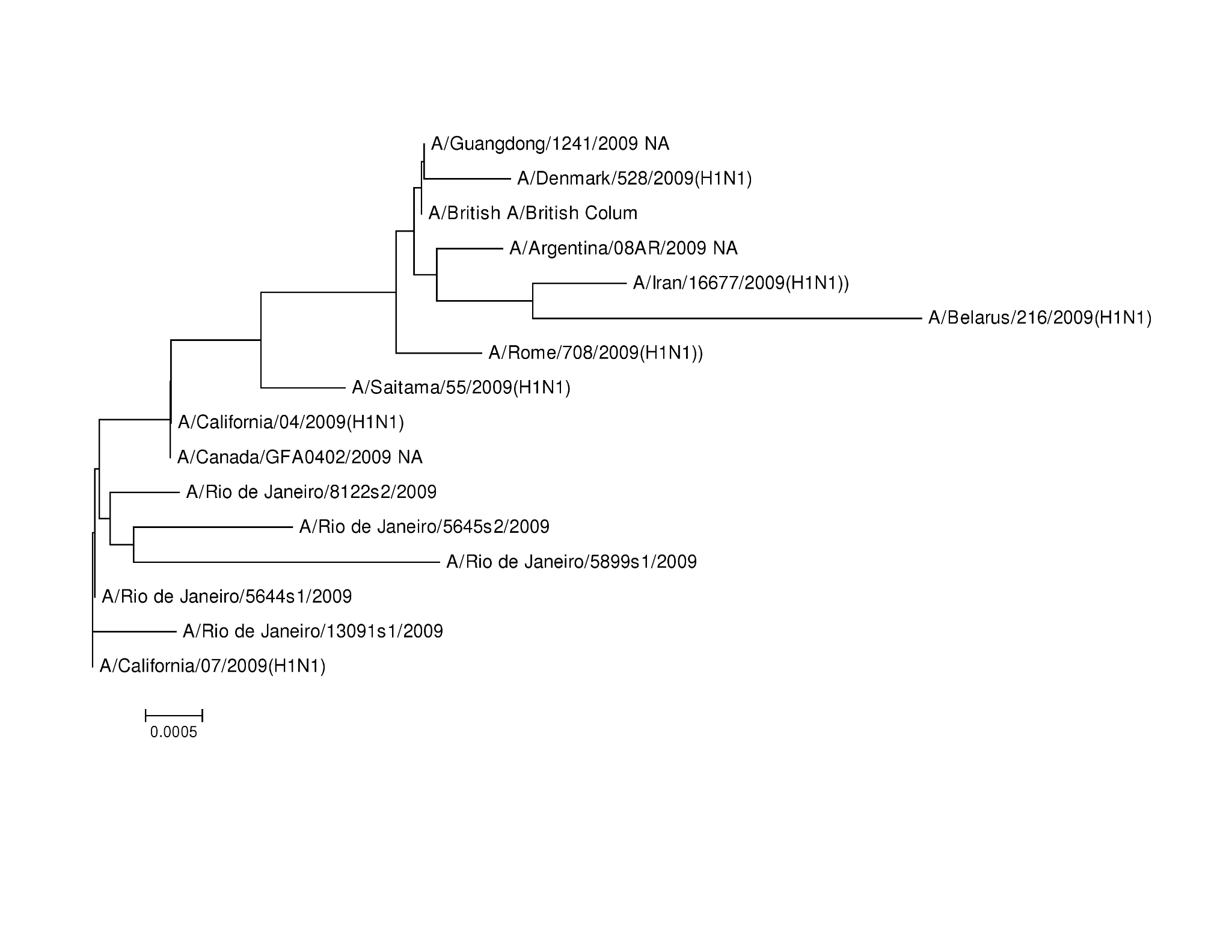

Supplement: Figure S2 — Phylogenetic tree of NA gene from the followed-up cohort. The bootstrap probability is not indicated for each interior branch since it is below 85%. The scale bar indicates the number of amino acid changes per site. The tree is rooted by California/07/2009 NA sequence. (0.18 MB TIF) [file pone.0014158.s015.tif]
